# Higher BMI and extraversion are associated with greater button-press force in a lab setting

**DOI:** 10.3389/fpubh.2025.1681360

**Published:** 2025-10-01

**Authors:** Baotian Chang, Songbin Yang, Nan Zhang

**Affiliations:** ^1^Beijing Key Laboratory of Green Built Environment and Energy Efficient Technology, Beijing University of Technology, Beijing, China; ^2^Mental Health Education and Counseling Center, Chongqing City Vocational College, Chongqing, China

**Keywords:** surface touch force, demographic, personality, surface transmission, film pressure sensor, touch behaviors

## Abstract

Surface transmission is a major route for gastrointestinal infections, with risk driven by human touch behaviors and microbial transfer rates. Greater touch force generally increases microbial transfer rates—a pattern supported by previous studies, which suggests that increased force may enhance the potential for surface transmission. This study aims to clarify individual differences in touch force and consider how these differences might relate to microbial transfer potential based on existing evidence. We recruited 115 participants and recorded force during two common touches—typing (complex) and elevator-button presses (simple)—using a touch-sensing device. Demographic attributes and personality traits of the participants were assessed through questionnaires. In simple touches, higher BMI (*r* = 0.35, 95% CI [0.17, 0.51], *p* < 0.01; Beta = 0.32, 95% CI [0.10, 0.54], *p* < 0.05) and extraversion (*r* = 0.21, 95% CI [0.02, 0.38], *p* < 0.05; Beta = 0.25, 95% CI [0.03, 0.47], *p* < 0.05) predicted greater force; no demographic attributes or personality variables influenced complex touches, and sex had no effect. In practical terms, individuals with higher BMI or extraversion may disproportionately contaminate—and be exposed to—high-touch surfaces.

## 1 Introduction

Surface transmission is not only a primary route for the spread of most gastrointestinal infections, but also a potential route for some respiratory infections (1–3). Many researchers explored the mechanisms through which human touch behaviors influence the risk of surface transmission. People touch their facial mucous up to 34.3 times per hour, indicating the high potential risk of surface transmission (4). The transfer rate of pathogens between hands and surfaces, is significantly affect the surface transmission. The transfer rate is known to be affected by touch behaviors (e.g., touch force), as demonstrated by a significant positive correlation between transfer rate and touch force (5). Higher transfer rates increase the risk of infection via surface transmission (6, 7). Therefore, understanding the impact of touch behaviors on surface transmission is important.

Previous studies on surface touch behavior focused on touch frequency and number of people touched the surface (4), and few studies considered the touch force. Surfaces are generally divided into private surfaces and public surfaces (8). Each surface type has its own specific touch patterns, such as touch type (e.g., grasping, pressing), touch frequency, and touch force. According to the complexity of operations, surface touch behaviors can be categorized into complex operations, also known as fine motor skills (9), and simple operations. Fine motor skills require finger control, body coordination, and concentration, with proficiency varying among individuals (10). In contrast, simple operations demand less precision and do not require significant focus or proficiency. Surface touch behaviors vary widely.

Individuals display substantial variation in their touch behaviors, influenced by both demographic attributes and personality traits. Younger individuals tend to exhibit higher activity levels and restlessness (11). Factors like body mass index (BMI), exercise frequency, and sleep duration also affect behavioral performance (12, 13). For instance, regular physical exercise can enhance individuals’ attention and memory, thereby improving their work efficiency in daily life. In contrast, individuals with insufficient sleep may exhibit more negative emotions during social interactions, which can adversely affect their interpersonal relationships (14). Empathetic individuals are more likely to follow epidemic prevention measures and may lead to lower infection risks (15), while those with a strong sense of entitlement are less compliant and face higher risks (16, 17). Psychological traits such as fairness perception, sense of power, and moral grandstanding significantly influence social behavior (18–20). However, there is a lack of understanding on the impact of demographic attributes and personality traits on touch behavior.

Grounded in the hypothesis that elevated touch force amplifies infection risk, this study investigates how demographic attributes (age, sex, BMI, exercise frequency, sleep duration) and personality traits (extraversion, empathy, entitlement, etc.) relate to the force exerted during complex (keyboard typing) versus simple (elevator button) touch behaviors. Using correlation and regression analyses, we quantify the contribution of touch force to these behaviors. The interdisciplinary design offers an empirical basis for identifying individuals at heightened risk of contributing to or acquiring pathogens during surface transmission.

## 2 Methods

### 2.1 Ethical approval

The study was approved by the Ethics Committee of Beijing University of Technology (BJUT-JGXY-03). All participants provided written informed consent (see Supplementary material A), and the study was conducted anonymously.

### 2.2 Study design

This was a single center and single blind trial to assess how demographic attributes and personality traits influenced human touch behaviors. The study purposefully withheld the objective of the study to participants to minimize the impact on their behaviors, thereby reducing possible testing bias that might arise from such awareness. This test was conducted in a laboratory room at Beijing University of Technology and all data were collected between November 2024 and January 2025.

We collected two types of common touch behaviors with high touch frequency in daily life (21). Typing on keyboards is considered a private and complex operation typically occurring in office and requires a certain level of skill and proficiency. In contrast, pressing elevator buttons is a simple and public behavior that does not require specific skills. This study focused on above mentioned touch behaviors, collecting touch force data during these behaviors to reveal their associations with both demographic attributes and personality traits.

**Figure 1:**
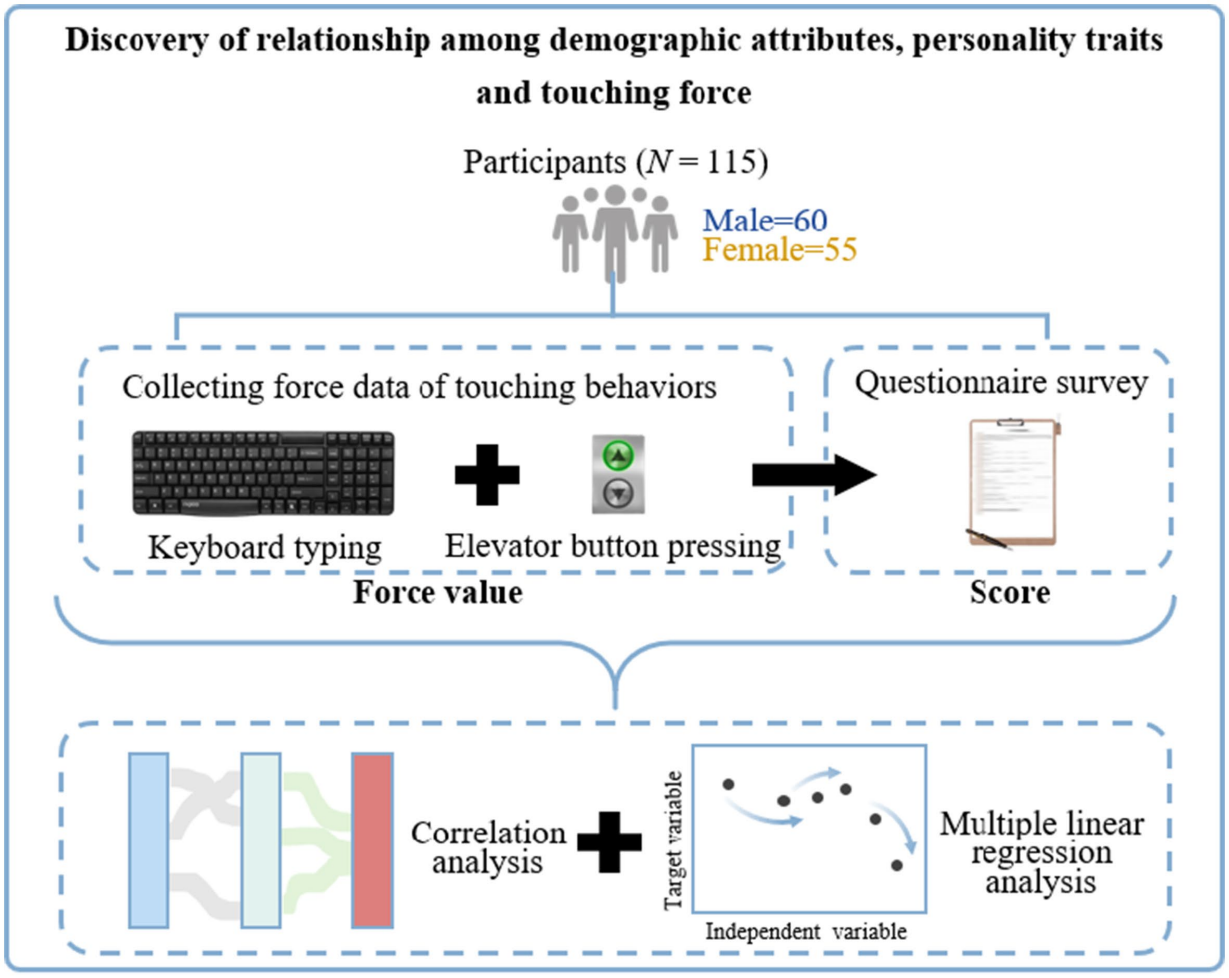
Testing framework of touch force, demographic attributes, and personality traits.

We recruited 115 participants to conduct formal test (Figure 1). Data on touch force were obtained from participants’ typing on keyboards and pressing elevator buttons. Data on demographic attributes and personality traits were collected through questionnaires and subjected to statistical analysis.

In the preliminary test, we verified the high consistency of the force values, regardless of the number of repeated measurements or the presence of environmental disturbances. Therefore, in the formal test, the impact of these factors on force data can be disregarded. For detailed description on the preliminary tests, see Supplementary material B.

### 2.3 Participant recruitment

The volunteers were eligible to participate if they could execute common touch-based activities, such as typing on keyboards or pressing elevator buttons. In the formal test, to determine an appropriate sample size, a G*power analysis (22) indicated that a minimum sample size of 101 participants was required. Oversampling to accommodate data loss, 117 participants were recruited on campus. Thus, we collected 115 qualified participants who have passed an attention check (see Supplementary material C; age *M* = 23.1, SD = 2.2; 60 males, 55 females).

### 2.4 Study procedures

The study was approved by the Ethics Committee of Beijing University of Technology (BJUT-JGXY-03). All participants provided written informed consent (see Supplementary material A), and the study was conducted anonymously.

#### 2.4.1 Touching behaviors collection

Surface touch force was recorded using a touch-sensing device, which aims to automatically collect human touch behavior data, like touch force, duration and frequency (23). The main components are list as follows: Rouxi piezoresistive thin film sensors (range = 0–6 N, d = 18 mm). Sensors were conditioned with an Arduino Mega 2,560 running at 20 Hz, low-pass filtered (second order Butterworth, cutoff frequency = 5 Hz), and calibrated with known weights before each session.

**Figure 2:**
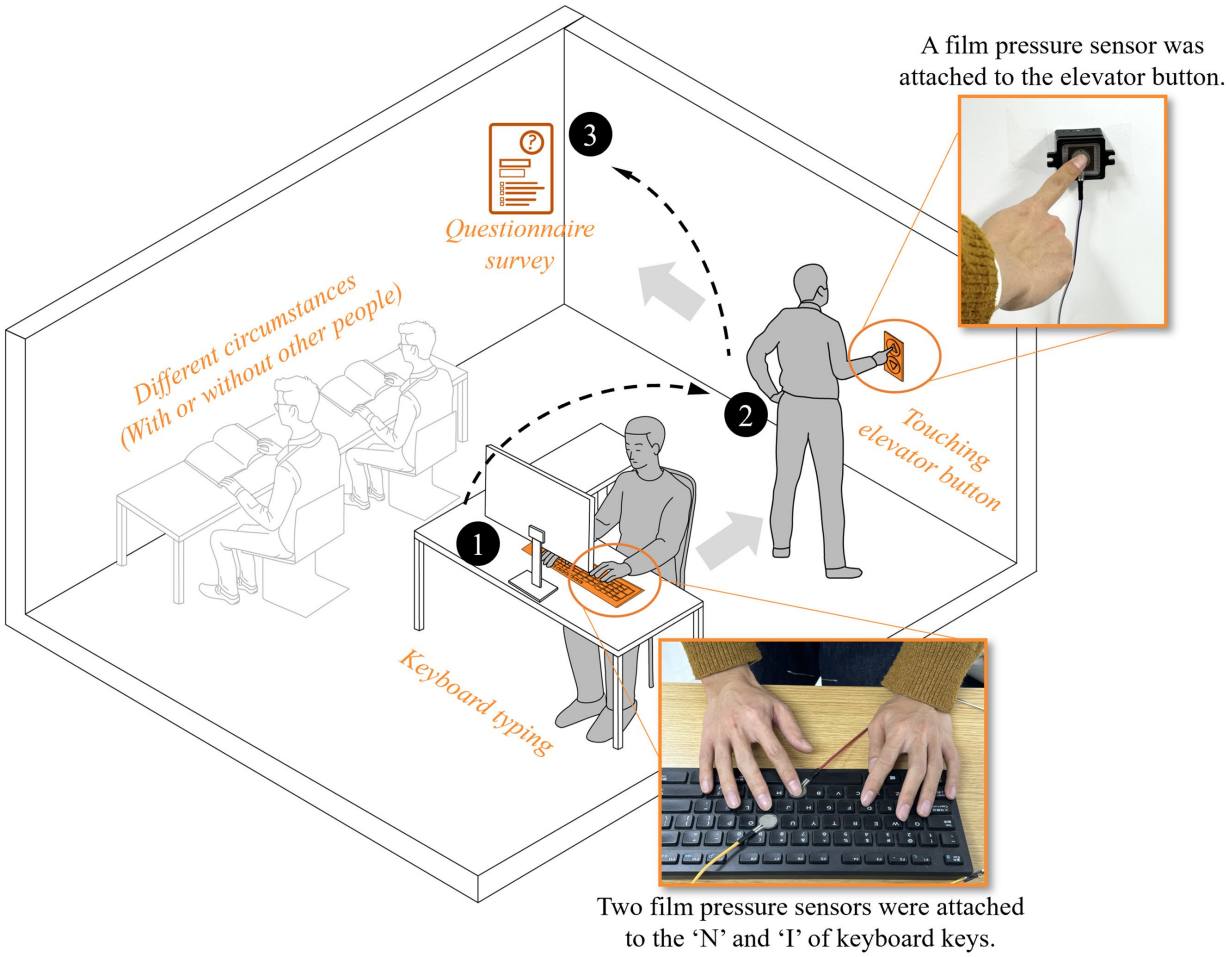
Schematic diagram of the testing procedure.

Prior to each test, the touch-sensing device was positioned and activated on designated keys of the keyboard and the elevator buttons (Figure 2). Participants were able to independently and successfully complete the two types of touch behaviors described above. In this test, the text input on the keyboard consisted of Chinese paragraphs. A pre-test of typing habits showed that the “N” and “I” keys were the most frequently touched and these keys were struck almost exclusively by the right index finger and middle finger, during standard Chinese text input. Given that excessive sensor equipment could potentially interfere with participants’ behaviors during the test, and to balance data yield against interference, only the “N” and “I” keys were instrumented with sensors.

During the test, the task order was fixed (keyboard typing first, elevator button pressing second) based on pilot data indicating negligible fatigue effects. Participants were required to use a designated desktop computer equipped with a keyboard fitted with touch-sensing device to perform the typing task. The touch force of every keystroke on the two instrumented keys was extracted and the individual’s keyboard force was defined as the mean across all valid strokes. The typing content was standardized Chinese text (specific operational instructions are detailed in Supplementary material D). A wall-mounted 25 mm × 25 mm square elevator button was fitted with the same sensor. Participants completed two warm-up presses followed by 10 test presses at 5-s intervals; the first two warm-up test presses were discarded. Tests with artefacts (force > 3 SD from the individual mean) were excluded. The remaining eight tests were averaged to yield the participant’s elevator-button force (Figure 2). In the button pressing testes, each participant was instructed to press the button ten times at 5-s intervals.

Upon completion of the tests, the touch behavior data collected were uploaded to a computer through a storage module and an Arduino controller for detailed analysis of the touch force data.

#### 2.4.2 Demographic attributes and personality traits

Both demographic attributes and personality traits were collected by questionnaire. For demographic attributes, participants provided basic information (sex, age, height and weight (BMI), level of education) and other variables, including health status, stress status, sleep duration, exercise frequency, monthly household income, and social stratification were also collected (Table 1).

**Table 1:** Variables used in study.

Variable type (number)	Parameter	Scale	Explanation
Demographic attributes (10)	Sex, age, height and weight (BMI), level of education, health status, stress status, sleep duration, exercise frequency, monthly household income, and social stratification	/	/
Personality traits (13)	Extraversion	International Personality Item Pool	*Extraversion* describes an individual’s level of activity and emotional expression in social settings. Individuals with high *extraversion* are typically cheerful and sociable (55).
	Agreeableness		*Agreeableness* reflects an individual’s cooperativeness and prosocial behavior in interactions with others. Highly agreeable individuals are usually friendly and helpful (55).
	Conscientiousness		*Conscientiousness* involves an individual’s self-discipline, organizational skills, and goal orientation. Highly conscientious individuals are typically orderly and reliable (56).
	Neuroticism		*Neuroticism* pertains to an individual’s emotional stability and reactivity. Those with high *neuroticism* are more prone to experiencing anxiety and tension (56).
	Openness		*Openness* reflects an individual’s receptiveness to new things, ideas, and experiences. Highly open individuals typically possess curiosity and creativity (57).
	Modesty	Brief HEXACO Inventory	*Modesty* describes an individual’s *modesty* and willingness to accept feedback. Highly *modesty* individuals are more open to others’ opinions and suggestions (58).
	Sincerity		*Sincerity* characterizes an individual’s authenticity in social interactions. Highly sincere individuals are more likely to express their true thoughts (58).
	Greed avoidance		*Greed avoidance* reflects an individual’s self-control in the face of material temptations. Those with high *greed avoidance* are less likely to excessively pursue material gains (59).
	Fairness		*Fairness* reflects an individual’s justice and selflessness in dealing with others. Highly fair individuals tend to consider others’ interests in decision-making (59).
	Entitlement	Psychological Entitlement Scale	*Entitlement* is characterized by an individual’s belief that they inherently deserve privileges or special treatment. High levels of psychological *entitlement* can lead to conflicts in interpersonal relationships (60)
	Empathy	Interpersonal Reactivity Index-C	*Empathy* refers to an individual’s ability to understand and feel the emotions of others. Highly empathetic individuals are more likely to exhibit behaviors such as helping others, cooperating, and showing understanding (61).
	Power	Power Scale	*Power* typically denotes an individual’s subjective perception of their influence and control in social interactions. Individuals with a high sense of *power* often display greater confidence and decision-making abilities (19).
	Moral grandstanding	Moral Grandstanding Motivation Scale	*Moral grandstanding* refers to an individual’s tendency to express moral views in public discussions to enhance their status or image in the eyes of others (62).
Touching behaviors (2)	Force of touching keyboards and elevator button	/	/

In addition to obtaining participants’ demographic attributes, the classic scales were administered for investigating personality traits, including the Big-Five personality factors (extraversion, agreeableness, conscientiousness, neuroticism and openness), honesty-humility (modesty, sincerity, greed avoidance, and fairness), entitlement, empathy, power and moral grandstanding (detailed description about scales are summarized in Supplementary material E). Reliability for each domain and aspects were adequate in all studies (min. *α* = 0.62). To examine the structural validity of all measurement scales within our sample, we conducted confirmatory factor analyses (CFA). The results, as detailed in Supplementary Table S1, indicated that all key fit indices met acceptable psychometric standards (24), demonstrating good construct validity for the employed scales in this study.

### 2.5 Statistical analysis

Given the exploratory nature of this study, our statistical analysis was designed to identify variables potentially associated with touch force and to generate hypotheses for future research. Descriptive statistical methods were employed to outline the basic characteristics of the participants. We calculated internal consistency statistics for scales (Supplementary material F; Supplementary Table S2), followed by Pearson correlation analysis with *p* values for both dependent variables (force of touching keyboards and elevator buttons) and independent variables (demographic attributes and personality traits). The Fisher’s r to Z transformation converts the Pearson correlation coefficient r to a normal distribution, allowing comparison of differences between two correlation coefficients (25, 26). It can be used to calculate and express the correlation differences between male and female samples using *p* values. In addition, p values for all exploratory variables were subjected to False Discovery Rate (FDR) correction using the Benjamini-Hochberg procedure (*Q* = 0.10).

Finally, this study employed multiple linear regression analysis to further explore the relationship between touch force and both demographic attributes and personality traits. We explicitly emphasize that this regression model is exploratory, and its results should be considered preliminary and heuristic. The value of the variance inflation factor (VIF) was less than 5, indicating that there was no significant issue of multicollinearity problem (27) (Supplementary material G; Supplementary Table S4). Specifically, multiple linear regression models were used to estimate the effect of both demographic attributes and personality traits on touching keyboards and elevator button, respectively. To examine the specific contribution of each variable to touching force, for each touching behavior, we followed Rengifo (28) and Furnham (29), first regressed touching force onto all thirteen personality traits simultaneously (Model 1). Next, we added demographic attribute variables (Model 2). The further hierarchical regression models were used to evaluate the effect of the selected independent variables on touching force value, thereby constructing models with stronger predictive performance.

A *p* value < 0.05, < 0.01 or < 0.001 were considered to be statistically significant. All data of demographic attributes and personality traits were collected using Tencent Questionnaire, and analyses were conducted using SPSS Statistics v26.0.

## 3 Results

We first examined the data with a correlation analysis of all the data of concern. Thereafter we did a series of regression analyses to test prediction performance of variables.

### 3.1 Descriptive statistics and correlation analysis

The descriptive statistics and bivariate Pearson correlations among study variables for processed force of touching keyboards and elevator buttons are presented in Supplementary Tables S1-3,5,6.

**Figure 3:**
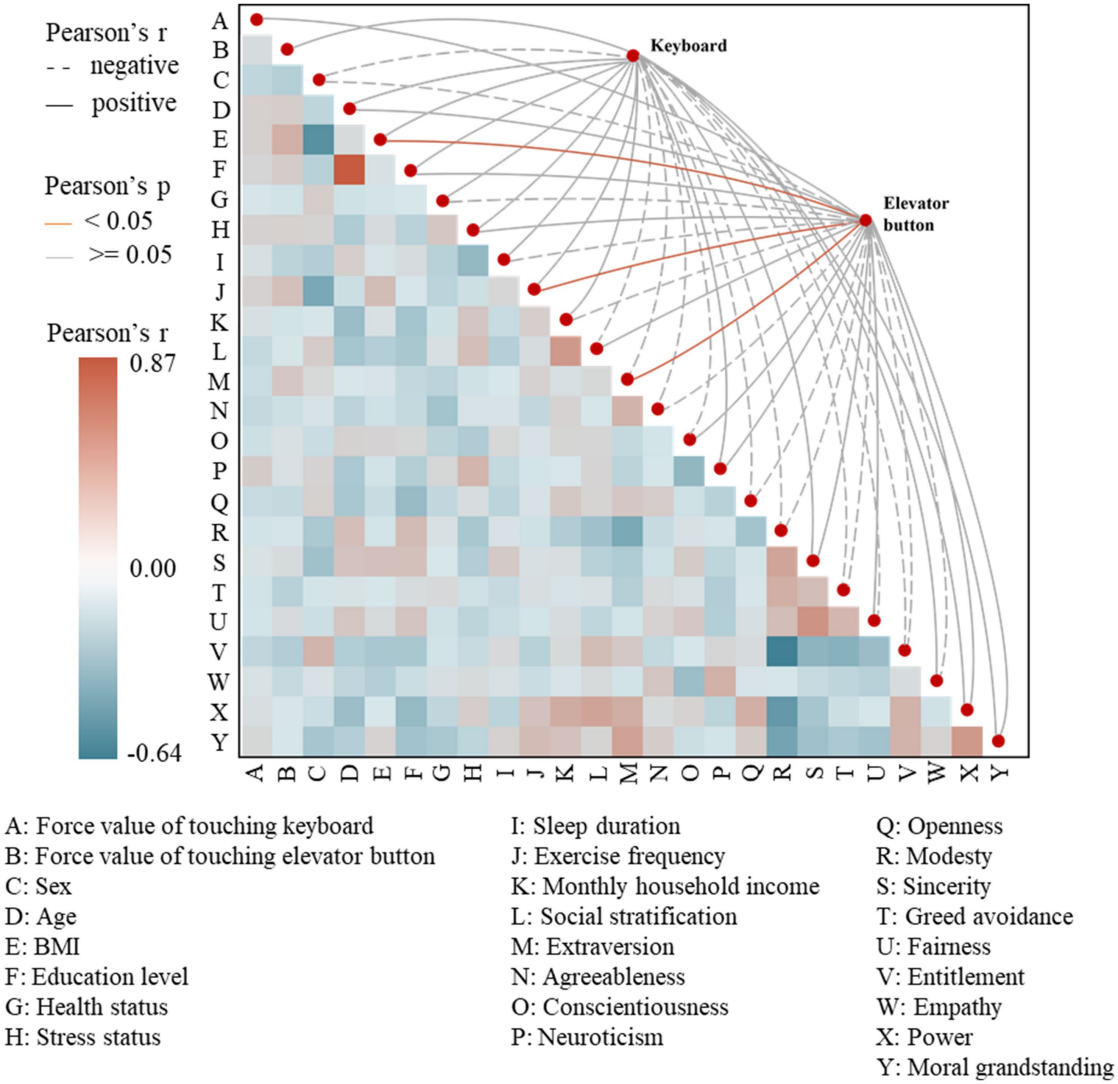
Heatmap of correlation analysis.

In this study, there was no significant correlation between force value on keyboards and elevator buttons (*r* = 0.08, 95% CI [−0.11, 0.26], *p* > 0 0.05). No personality traits or demographic attributes showed significant correlations with touch force on keyboards. In contrast, BMI, exercise frequency, and extraversion were significantly positively correlated with touch force on elevator buttons (Figure 3). Specifically, the highest significant correlation was found between BMI and touch force on elevator buttons (*r* = 0.35, 95% CI [0.17, 0.51], *p* < 0.01), indicating a strong positive relationship. Exercise frequency (*r* = 0.25, 95% CI [0.06, 0.42], *p* < 0.01) and extraversion (*r* = 0.21, 95% CI [0.02, 0.38], *p* < 0.05) also showed positive correlations with touch force on elevator buttons. Additionally, among these three correlated variables (BMI, exercise frequency, and extraversion), a significant positive correlation was observed between BMI and exercise frequency (*r* = 0.27, 95% CI [0.08, 0.44], p < 0.01), while extraversion did not show significant correlations with the other two variables.

Given the balanced sample sizes for male and female participants, we conducted exploratory, hypothesis-generating analyses by stratifying the correlation analyses by sex. It is important to note that these analyses were severely underpowered and subjected to a high number of multiple comparisons; therefore, any findings should be interpreted with caution and require independent replication. For both keyboard touch force and elevator button touch force, it can be concluded that sexes do not have a significant impact on touch behavior (*p* > 0.05).

For keyboard touch force, no significant correlations were found between both demographic attributes and personality traits, and touch force for either males or females. Among males, lower agreeableness and higher neuroticism were associated with greater keyboard touch force. However, these personality traits did not show significant correlations with touch force in female samples. Conversely, lower modesty and higher power were associated with greater keyboard touch force in female participants. Among all studied variables, only fairness showed significant correlations in both male and female samples. Specifically, lower fairness scores in males and higher fairness scores in females were associated with greater keyboard touch force. This phenomenon may be related to gender socialization differences. Critically, after applying False Discovery Rate (FDR) correction for multiple comparisons across all exploratory tests, none of these correlations remained statistically significant (all q > 0.10). This confirms that these isolated findings are not robust on their own and serves primarily to generate hypotheses for future research with dedicated, larger samples.

### 3.2 Regression analysis

Multiple linear regression analyses were conducted separately for keyboard touch force and elevator button touch force, with each serving as the dependent variable. All variables except extraversion, entitlement and BMI exhibited consistent null effects across two typical touching behaviors. For keyboard touch force, regression results showed that entitlement explained 11% of touching force variance (adjusted *R*^2^ = 0.11, 95% CI [0.02, 0.22], *p* < 0.05), and showed a negatively significant prediction (Beta = −0.26, 95% CI [−0.44, −0.08], *p* < 0.05) for touching force of keyboard. Albeit, demographic attribute variables were entered into the model with the predictor of touching force disappearing (Model 2, Table 2), which demonstrates that the predictive capacity of entitlement is susceptible to interference from the demographic attribute variables.

**Table 2:** Regression analyses for variables predicting touching force.

Variables	Keyboard		Elevator button	
	Beta (95% CI)		Beta (95% CI)	
	Model 1	Model 2	Model 1	Model 2
Extraversion	−0.09 (−0.27, 0.09)	−0.15 (−0.33, 0.03)	0.29* (0.05, 0.53)	0.25* (0.03, 0.47)
Agreeableness	−0.09 (−0.27, 0.09)	−0.05 (−0.23, 0.13)	−0.12 (−0.30, 0.06)	−0.06 (−0.24, 0.12)
Conscientiousness	−0.01 (−0.19, 0.17)	−0.02 (−0.20, 0.16)	0.06 (−0.12, 0.24)	0.02 (−0.16, 0.20)
Neuroticism	0.20 (−0.04, 0.44)	0.22 (−0.02, 0.46)	0.11 (−0.07, 0.29)	0.03 (−0.15, 0.21)
Openness	−0.09 (−0.27, 0.09)	−0.08 (−0.26, 0.10)	−0.11 (−0.29, 0.07)	−0.07 (−0.25, 0.11)
Modesty	−0.15 (−0.33, 0.03)	−0.13 (−0.31, 0.05)	−0.12 (−0.30, 0.06)	0.03 (−0.15, 0.21)
Sincerity	0.08 (−0.10, 0.26)	0.04 (−0.14, 0.22)	0.09 (−0.09, 0.27)	0.03 (−0.15, 0.21)
Greed avoidance	−0.02 (−0.20, 0.16)	0.01 (−0.17, 0.19)	−0.18 (−0.36, 0.00)	−0.20 (−0.38, −0.02)
Fairness	0.01 (−0.17, 0.19)	−0.04 (−0.22, 0.14)	0.06 (−0.12, 0.24)	0.06 (−0.12, 0.24)
Entitlement	−0.26* (−0.44, −0.08)	−0.21 (−0.39, 0.03)	−0.32* (−0.58, −0.06)	−0.14 (−0.32, 0.04)
Empathy	−0.01 (−0.19, 0.17)	0.01 (−0.17, 0.19)	−0.05 (−0.23, 0.13)	−0.01 (−0.19, 0.17)
Power	0.10 (−0.08, 0.28)	0.19 (−0.01, 0.39)	0.04 (−0.14, 0.22)	0.00 (−0.18, 0.18)
Moral grandstanding	0.17 (−0.05, 0.39)	0.13 (−0.07, 0.33)	−0.03 (−0.21, 0.15)	−0.05 (−0.23, 0.13)
Sex	/	0.09 (−0.09, 0.27)	/	0.11 (−0.07, 0.29)
Age	/	0.34 (−0.08, 0.76)	/	−0.01 (−0.19, 0.17)
BMI	/	0.06 (−0.12, 0.24)	/	0.32* (0.10, 0.54)
Education level	/	−0.10 (−0.28, 0.09)	/	0.15 (−0.05, 0.35)
Health status	/	0.00 (−0.18, 0.18)	/	−0.03 (−0.21, 0.15)
Stress status	/	0.08 (−0.10, 0.26)	/	0.10 (−0.08, 0.28)
Sleep duration	/	0.05 (−0.13, 0.23)	/	−0.10 (−0.28, 0.08)
Exercise frequency	/	0.07 (−0.11, 0.25)	/	0.19 (−0.01, 0.39)
Monthly household income	/	0.09 (−0.09, 0.27)	/	−0.05 (−0.23, 0.13)
Social stratification	/	−0.16 (−0.34, 0.02)	/	0.07 (−0.11, 0.25)
*R* ^2^	0.11	0.19	0.17	0.31

Model 1: Only personality trait variables are considered; Model 2: All independent variables are considered.

*p < 0.05.

The bold values presented in table denote the statistically significant variables within the model.

For elevator button touch force, extraversion was notably the strongest positive predictor of touching force, being robust to the inclusion of all the variables in elevator button (Model 1 and 2, Table 2). When only the 13 personality trait variables were considered, extraversion and entitlement showed strong predictive performance for touch force. Extraversion had a positive predictive ability (Beta = 0.29, 95% CI [0.05, 0.53], *p* < 0.05), while entitlement had a negative predictive ability (Beta = −0.32, 95% CI [−0.58, −0.06], *p* < 0.05). Model 2 demonstrated that after including demographic attribute variables, the significance of extraversion was retained, and its relative influence on touch force for elevator buttons (Beta = 0.25, 95% CI [0.03, 0.47], *p* < 0.05) remained nearly consistent with Model 1. After considering demographic attributes, the significant negative predictive role of entitlement was no longer significant, and BMI showed a significant positive predictive ability (Beta = 0.32, 95% CI [0.10, 0.54], *p* < 0.05). In the regression model, exercise frequency did not emerge as a significant predictor, indicating that its bivariate association with touch force was largely accounted for by BMI and extraversion. The predictive ability of entitlement also showed that it is easily interfered with by other independent variables. This indicates that entitlement cannot be used as an independent predictive indicator for touch force.

Given the inability to identify reliable significant predictors when conducting multiple linear regression analysis on the dependent variable of touching keyboard force values, a further hierarchical regression analysis was carried out using force values of touching elevator button as the criterion variable. BMI was significant predictor of force value of touching elevator button, accounting for 12.3% of variance (Table 3). In step 2, extraversion was entered into the equation. This showed that both BMI and extraversion were significant predictors of force values, which in addition accounted for 4.1% of variance. It revealed that among the study variables, both BMI and extraversion were the positive predictor of force value of touching elevator button (Beta = 0.35, 95% CI [0.18, 0.52], *p* < 0.001; Beta = 0.20, 95% CI [0.02, 0.38], *p* < 0.05). The final regression explained 16.4% of the variables of the outcome variable.

**Table 3:** Hierarchical regression analyses for variables predicting touching force on elevator button.

Variables	*B*	SE (B)	Beta (95% CI)	*R* ^2^	Δ*R*^2^
Step 1				0.123***	0.123***
BMI	0.05***	0.01	0.35 (0.18, 0.52)		
Step 2				0.164*	0.041*
BMI	0.05***	0.01	0.35 (0.18, 0.52)		
*Extraversion*	0.11*	0.05	0.20 (0.02, 0.38)		

*p < 0.05, ***p < 0.001.

## 4 Discussion

This study utilized a touch-sensing device based on film pressure sensors to collect 115 samples of touch force data and quantitatively assessed demographic attributes and personality traits through questionnaires. The study revealed which personal factors affect individuals’ daily surface touch force.

This study found that individuals with higher levels of extraversion and greater BMI exhibited higher force values when touching elevator buttons. Extraversion was significantly positively correlated with touch force on elevator buttons (*r* = 0.21, 95% CI [0.02, 0.38], *p* < 0.05) and independently predicted touch force (Beta = 0.25, 95% CI [0.03, 0.47], *p* < 0.05). This may be because extroverted individuals typically exhibit optimistic and proactive personality traits and tend to seek novel experiences and take risks (30), leading them to exert greater force when performing simple actions such as touching elevator buttons. Additionally, elevator button-touching behavior carries certain social attributes (31). Extroverted individuals generally have a stronger ability to adapt to external environments and place greater emphasis on social networks (32). Previous studies suggested that agreeableness in the Big Five personality traits is associated with a tendency for exploration, potentially increasing the risk of pathogen exposure, whereas individuals with low conscientiousness and low neuroticism tend to engage in more cautious behaviors, thereby reducing infection risk (33). However, this study found no significant correlation between surface touch force and personality traits other than extraversion. This indicated that different personality traits may have varying impacts on behaviors related to infectious disease transmission. For instance, while extraversion significantly influences touch force, conscientiousness plays a greater role in adherence to preventive measures. This finding further supports the multidimensional and heterogeneous effects of personality traits on individual behavior.

BMI is another trait that is significantly associated with touch force on elevator buttons and positively predicts touch force (*r* = 0.35, 95% CI [0.17, 0.51], *p* < 0.01; Beta = 0.32, 95% CI [0.10, 0.54], *p* < 0.05). BMI is a key indicator of the weight-to-height ratio and is commonly used to assess an individual’s degree of obesity. According to the World Health Organization (WHO) standards, a BMI of ≥30 kg/m^2^ is classified as obesity, while a BMI between 25 and 30 kg/m^2^ is considered overweight (34). Study proved that individuals with higher BMI may possess greater muscular strength, particularly in the arms and hands. This strength advantage may lead them to exert greater force unconsciously when touching surfaces (35). Additionally, individuals with obesity tend to have thicker subcutaneous fat, which can affect muscle flexibility and tactile feedback (36, 37). As a result, they may need to apply a greater force to achieve the same level of tactile sensation when touching surfaces (38). Although exercise frequency showed a positive correlation with elevator button force, this effect was no longer significant after adjusting for BMI and extraversion. Nevertheless, the bivariate association hints at a potential mechanism: individuals who exercise more often may possess greater finger-hand strength (39) or adopt a more vigorous motor style, leading momentarily to higher touch forces on high-touch surfaces such as elevator buttons. Greater force could increase the area and depth of finger-surface touch, potentially enhancing the transfer of skin-resident microorganisms to the surface (40). However, because BMI and extraversion accounted for the shared variance, exercise frequency is unlikely to represent an independent risk factor in our samples. Future studies with larger populations and direct microbial quantification are needed to clarify whether habitual physical activity modulates fomite force-mediated transmission risk.

It is known that extroverted individuals, due to their strong social tendencies and frequent participation in close-contact interactions, face a higher risk of respiratory infectious disease transmission through the airborne or droplet route (41). Similarly, individuals with higher BMI may be more susceptible to severe outcomes from respiratory and gastrointestinal infections due to comorbidities (42, 43). Our study introduces a novel behavioral dimension to this picture: higher touch force. Previous research has established that greater applied force can lead to a higher transfer rate of pathogens between surfaces and fingers, thereby potentially increasing the microbial exposure per touch event (44). Therefore, the stronger pressing behavior we observed in high-BMI and extroverted individuals could represent a previously overlooked mechanism that might contribute to their overall exposure profile, specifically for surface-mediated transmission routes. However, it is critical to emphasize that our study did not measure microbial transfer, exposure dose, or health outcomes. The overall risk of infection via surface transmission is a complex function of numerous factors, including touch frequency, touch duration, contact area, surface properties, and environmental conditions (4). Thus, our findings should not be interpreted as evidence that these groups have a higher infection risk, but rather that they exhibit a behavior (high touch force) that, based on biomechanical principles, could be integrated as a variable into future quantitative microbial risk assessment (QMRA) models to improve their predictive accuracy (45). A key strength of this study is its exploratory design, which identified candidate factors such as BMI and extraversion that may be associated with touch force from a wide array of variables. However, it must be noted that despite employing FDR correction and limiting the number of predictors in the regression model 1, these findings remain susceptible to false positives (46). Consequently, our conclusions should be considered heuristic, offering specific targets and a clear direction for future validation studies.

In this study, sex was found not significantly correlated with touch force and did not effectively predict touch force. However, sex may have a more significant influence on other behavioral factors. Previous studies suggested that males tend to be more reserved, less expressive of emotions, and more inclined toward team activities and competitive games (47). In contrast, females are generally more emotionally expressive, place greater emphasis on emotional connections, and build interpersonal relationships through communication (48). In this study, with a balanced sex ratio among participants, an independent analysis of different sexes revealed correlations between personality traits—such as agreeableness, neuroticism, modesty, power, and fairness—and both keyboard typing force and elevator button touch force. However, these correlations were not observed in the overall sample analysis, likely because they are dependent on sex factors and thus fail to independently predict touch force or establish stable correlations at the overall sample level.

This study found that certain demographic attributes and personality traits were associated with surface touch force, but probably only in the context of simple touch behaviors. The independent variables exhibited different levels of influence when predicting these two types of behavior, which may be attributed to several factors. On the one hand, keyboard typing is a psychomotor skill that relies on proficiency, concentration, finger control, and bodily coordination while involving complex cognitive processes (9, 49). In contrast, pressing an elevator button is a simpler action with lower precision requirements and fewer interfering factors, making it more susceptible to individual traits and more predictable. For instance, operational proficiency significantly affects typing performance but has no notable impact on elevator button pressing in unprepared conditions (50). On the other hand, keyboard typing, as a fine motor skill (51), often requires individuals to adjust touch force according to task demands. This goal-directed nature encourages individuals to adopt more consistent touch strategies in complex touch behaviors, thereby significantly reducing individual differences in touch force (52). In summary, individual differences in touch force are more pronounced in simple touch behaviors, likely because such behaviors directly reflect an individual’s inherent physiological and psychological traits. In contrast, in complex touch behaviors, the combined effects of goal-directedness, skill level, and concentration diminish individual differences in touch force.

Based on the findings of this study, the following directions for future research may be proposed. First, the behavioral traits identified (high BMI and extraversion) could be investigated as potential predictors in epidemiological studies of surface-transmitted infections. Second, incorporating touch force patterns related to BMI and personality into computational transmission models is recommended to theoretically test their impact on infection risk. Finally, if further evidence solidifies this link, raising awareness among individuals who exhibit strong pressing behavior could be explored as a component of hygiene strategies. The ultimate goal is to move toward more personalized and effective public health recommendations.

This study had several limitations. First, the participants were recruited from the university, which means the sample may not represent the broader population of individuals (such as age or education level), potentially limiting the generalizability of our findings. Second, this study focused on limited demographic attributes and personality traits, as these factors are typically associated with human behaviors according to the literature. This does not mean that there is no correlation between the factors not discussed in this article and touch behavior. A longitudinal study on the determinants of self-efficacy revealed that these factors undergo certain changes over time (53). Then, the contact area during touches was not measured, which is a critical variable governing force and microbial transfer rate. Only two keyboard keys were instrumented, and elevator buttons represent a single type of push-button, limiting the generalizability across all surface-touch behaviors. Third, the exploration of numerous variables, while yielding interesting correlates, involved extensive multiple testing, and the final regression models explained a modest proportion of the variance (R^2^). Although our single blind design reduced demand characteristics, withholding the true study purpose may have introduced alternative sources of variability, such as momentary affect and idiosyncratic task interpretations. We did not collect post-task measures of mood or comfort, and therefore cannot empirically rule out their influence. Future studies should include brief affect/comfort ratings or employ partial-deception protocols to better disentangle these potential confounds while maintaining experimental realism. Finally and most importantly, this study did not measure any microbiological outcomes; we cannot confirm that higher forces led to greater microbial transfer or exposure in our test. To build on our findings, future studies should engage broader community samples, follow participants over time to capture evolving psychological and contextual influences, record touch force across varied everyday surfaces, and embed momentary affect measures while contrasting single-blind, partial-deception, and fully informed protocols to isolate trait-driven effects from methodological aspect. In addition, future studies could apply a published force-vs-transfer efficiency function to the distribution of forces we measured. For example, under the transfer function from Xie et al. (54), the 0.8 N mean difference between the highest and lowest BMI quartiles translates to an estimated 6% increase in microbes transferred per button press—an illustrative figure that awaits empirical validation. This would allow, under stated assumptions about pathogen concentration and contact area, an estimation of the relative change in the mass of microbes transferred per touch event for different behavioral phenotypes. Our data provide the necessary behavioral input parameters for such a model.

## 5 Conclusion

Greater force on everyday surfaces such as elevator buttons increases the likelihood that microbes are transferred from fingers to shared objects and vice-versa. Our findings suggest that BMI and extraversion are two readily identifiable factors associated with heavier contact. In practical terms, individuals with these characteristics may unknowingly contribute more microbial load to high-touch surfaces, while also incurring a higher personal risk of picking up pathogens. Promoting lighter button contact or contactless control could therefore become an easy-to-adopt behavioral target for infection-prevention campaigns. Therefore, we suggest that building managers can install touch-free control devices or transform buttons with low-voltage activation technology to reduce the required strength. Public health institutions may customize health information-for example, “gently press and disinfect immediately after”-especially in fitness centers or social places where the high BMI and extroverts are common. Additionally, designers of daily devices can integrate tactile feedback to signal successful activation with minimal force, thus promoting all users to develop gentler touch habits.

## Data Availability

The raw data supporting the conclusions of this article will be made available by the authors, without undue reservation.
