# Supplementary material for: Higher BMI and extraversion are associated with greater button-press force in a lab setting
Source: Front Public Health. 2025 Oct 1;13:1681360. doi: 10.3389/fpubh.2025.1681360 (PMC12521194; doi:10.3389/fpubh.2025.1681360)
Supplement: Supplementary file 1 [file Supplementary_file_1.docx]

Supplementary Material

# Supplementary Material A.

# Informed Consent

Dear Participant:

Hello! We are very grateful for your participation in this test. In order to better understand your background information and ensure the accuracy of the testing data, we need you to fill in the following basic information. Please rest assured that all information will be strictly confidential and used solely for research purposes.

*Consent Statement*

This test mainly consists of two parts: (1) Completing keyboard typing and mouse clicking operations according to the Instructions for Operation. During the test, a film sensor system will be used to measure the contact force of the hands, with an estimated duration of 15 minutes; (2) Filling out a professional test questionnaire. The questions included are derived from psychology-related questionnaires and are designed to comprehensively obtain information about personal traits and behavioral habits. The questionnaire will be distributed and completed electronically on-site, with an estimated duration of 10 minutes.

Your responses will be strictly confidential. Research data will be collected and stored anonymously to ensure that your personal information cannot be identified. If you have any questions about the study or feel any discomfort during participation, please contact the research team (Person in Charge: Chang, Phone: 157****5737).

I have read the above information and voluntarily agree to participate in this test. I consent to the use of the information I provide for research purposes and confirm that the information I fill in is true and accurate.

Participant’s Signature: ______________________

Date: ____ Year ____ Month ____ Day.

# Supplementary Material B.

# Preliminary Test - Data Consistency Test

The study included two preliminary tests designed to assess the consistency of touch force data collected by the force film sensor-based touch-sensing device (Fig. S1). Specifically, Preliminary Test 1 aimed to eliminate the impact of temporal differences on human surface touch behavior, while Preliminary Test 2 sought to rule out the influence of environmental factors on surface touch behavior, thereby providing a solid basis for the formal test.


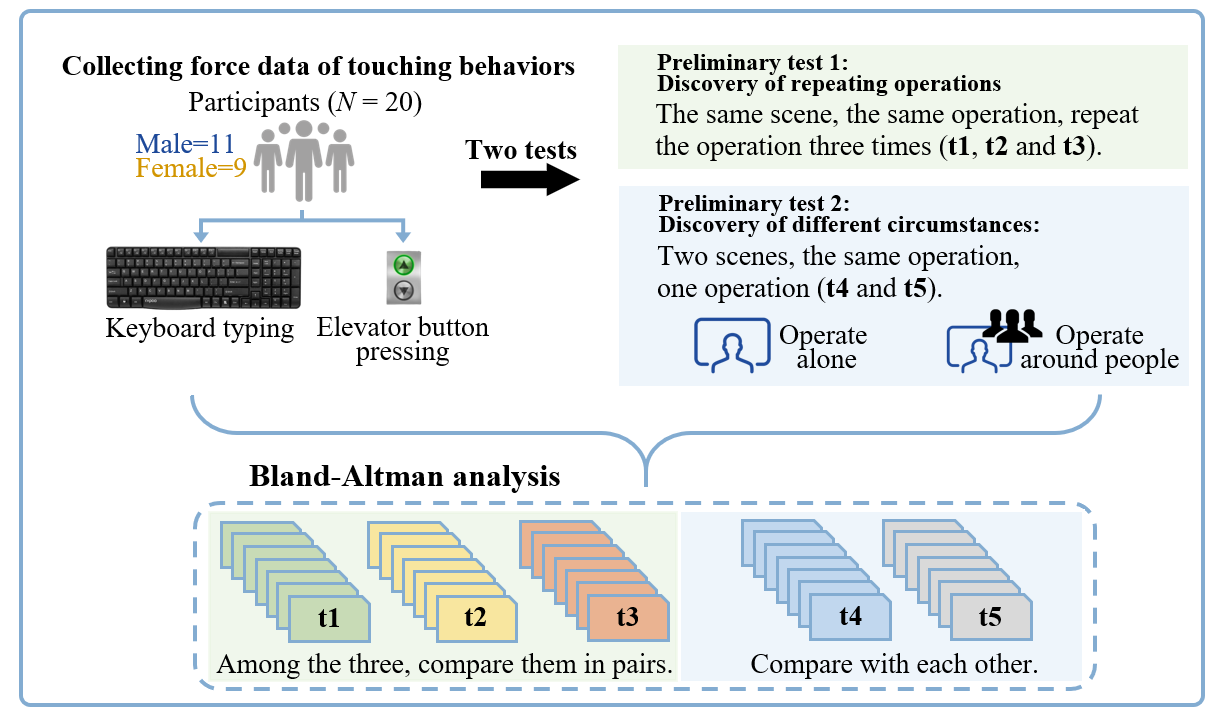


**Figure S1.** Preliminary test framework of touch behavior-individual trait.

In Preliminary Test 1, 20 participants were recruited to complete a set of typing on keyboards and pressing elevator buttons on three separate dates. Force values were collected using the touch-sensing device and were denoted as t1, t2, and t3 for each set of measurements. In Preliminary Test 2, the same participants performed the same touch operations under two different environmental conditions: with no other people present in the laboratory and with other people present. Force values were collected under these two conditions and were denoted as t4 and t5, respectively.

The Bland-Altman analysis was employed to evaluate the consistency and stability of data across different test samples [1]. The Bland-Altman plot provides a visual representation of the agreement between two measurements taken at different time points [2], thereby offering a reliable basis for analyzing preliminary test data and drawing definitive conclusions. In this study, we conducted pairwise comparisons of t1, t2, and t3, as well as t4 and t5.

The Bland-Altman plots for the preliminary tests (t1~t5) are shown in Figure S2. Nearly all sample points fall within the 95% limits of agreement, demonstrating strong consistency between the different tests. A mean difference (–0.07 N; 95 % LoA –0.33 to 0.21 N) of Situation A (Keyboard) and mean difference (0.07 N; 95 % LoA –0.40 to 0.65 N) of Situation B for Bland–Altman analysis indicates that force values maintain a high degree of consistency regardless of the number of repeated measurements or the presence of environmental disturbances, with no randomness observed. The results indicate that force values maintain a high degree of consistency regardless of the number of repeated measurements or the presence of environmental disturbances, with no randomness observed. Therefore, in the formal test, the influence of these factors on the force data can be disregarded.


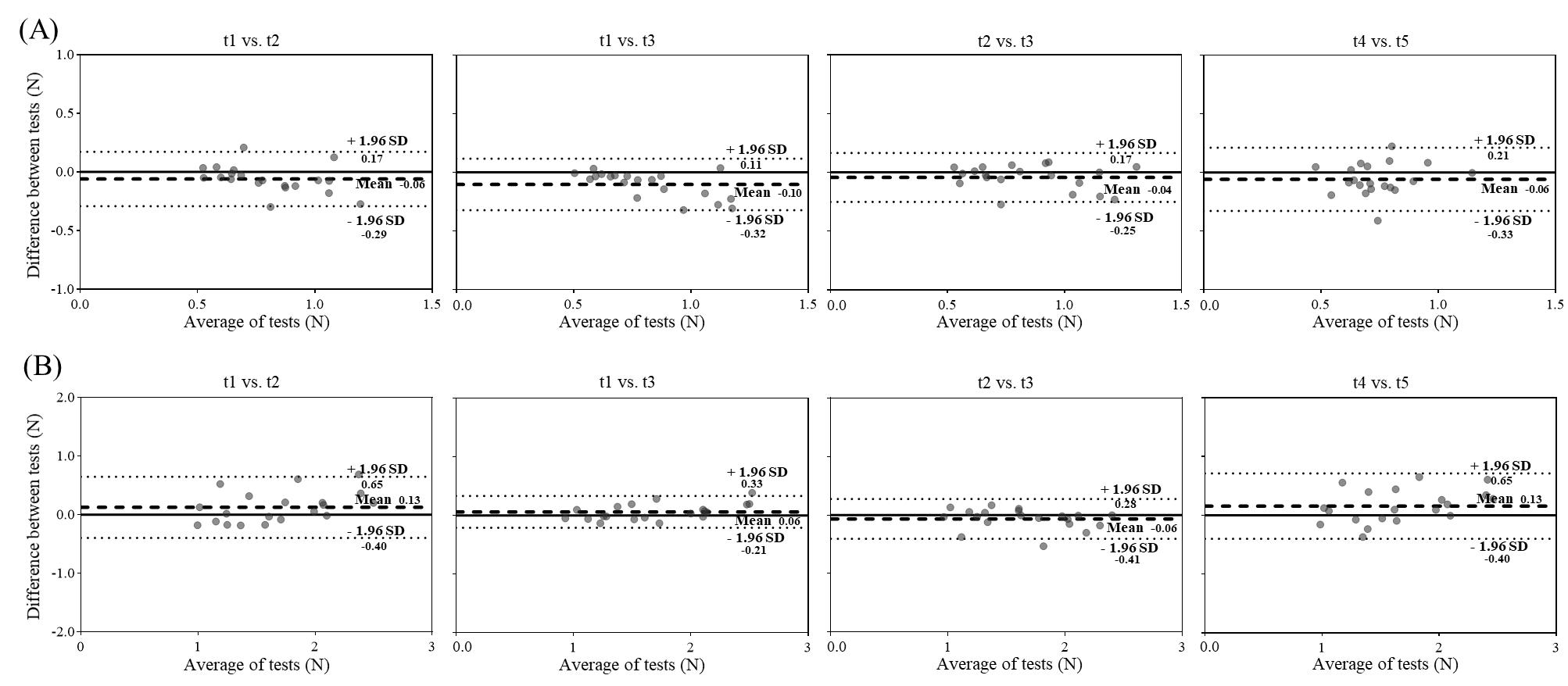


**Figure S2.** Bland-Altman plots of touching behavior force values: (A) Keyboard; (B) Elevator button, in which t1, t2 and t3 belong to preliminary test 1, t4 and t5 belong to preliminary test 2. (Dotted lines denote “limits of agreement” (average difference ± 1.96 × SD of the differences); SD: standard deviation).

# Supplementary Material C.

# Questionnaire - Basic Information

**Q1: Your gender:**

□ Male □ Female

**Q2: Your age:**

| Age | 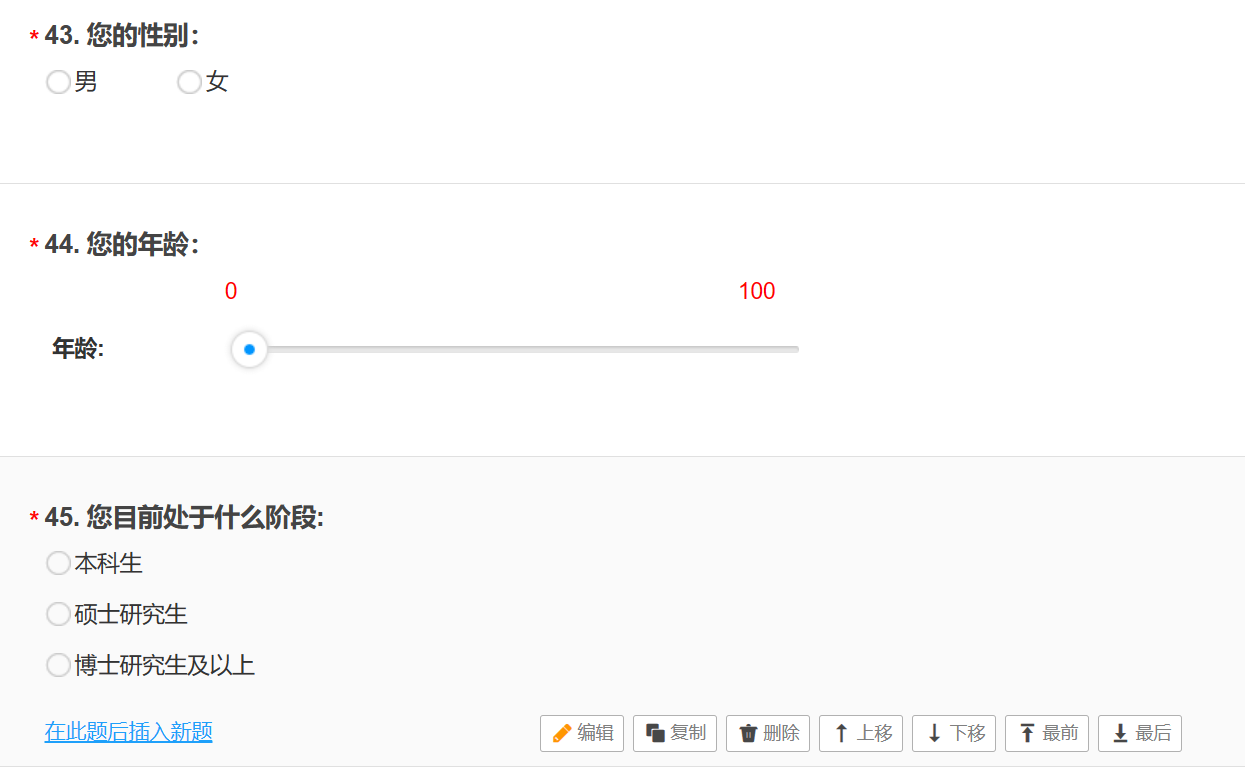 |
| --- | --- |

**Q3: Your height (cm):**

| Height | 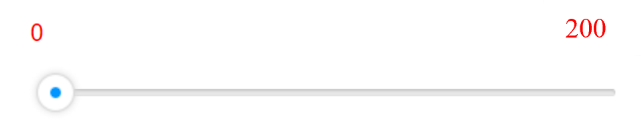 |
| --- | --- |

**Q4: Your weight (kg):**

| Weight | 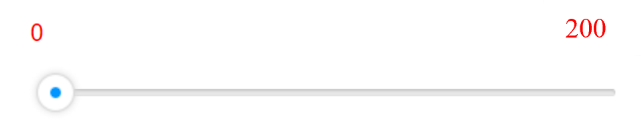 |
| --- | --- |

**Q5: Your current learning stage is:**

□ Undergraduate □ Master’s student □ Doctoral student

**Q6：Your current health status is:**

□ Good □ Fair □ Poor

**Q7：Your current stress status is:**

□ Never □ Less than once a week □ 1-2 times a week

□ 3-4 times a week □ 5 times or more a week

**Q8：Your sleep duration is:**

□ Less than 6 hours □ 6-7 hours □ 7-8 hours □ More than 8 hours

**Q9：Your exercise frequency is:**

□ Almost 0 □ 1-3 times per week □ 4-6 times per week

**Q10：Your monthly household income is:**

□ Less than 5,000 yuan □ 5,000 yuan to 9,999 yuan

□ 10,000 yuan to 14,999 yuan □ 15,000 yuan to 24,999 yuan

□ 25,000 yuan to 49,999 yuan □ More than 50,000 yuan

**Q11：Your social stratification is:**

Imagine that the ladder on the left represents the different social strata of families in China. The bottom of the ladder (01) represents the lowest social stratum, where people from these families have the most difficult living conditions, the lowest levels of education, the least desirable jobs, and the lowest incomes. The top of the ladder (10) represents the highest social stratum, where people from these families have the most favorable living conditions, the highest levels of education, the most prestigious jobs, and the highest incomes. Where do you think you are on this ladder? _____ (Please choose a number from 1 to 10).


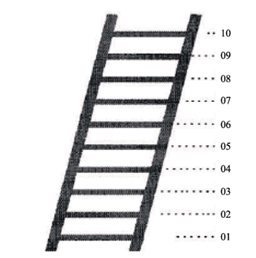


# Supplementary Material C.

# Questionnaire - Personality Traits Scale

Please respond to the following items using the number that best reflects your own beliefs. Please use the following 5-point scale:

1 = strong disagreement.

2 = moderate disagreement.

3 = neither agreement nor disagreement.

4 = moderate agreement.

5 = strong agreement.

(* The Mini-IPIP of Big Five Trait *: Extraversion, agreeableness, conscientiousness, neuroticism, openness)

1. Am the life of the party.

2. Sympathize with others’ feelings

3. Get chores done right away.

4. Have frequent mood swings.

5. Have a vivid imagination.

6. Don’t talk a lot.

7. Am not interested in other people’s problems.

8. Often forget to put things back in their proper place.

9. Am relaxed most of the time.

10. Am not interested in abstract ideas.

11. Talk to a lot of different people at parties.

12. Feel others’ emotions.

13. Like order.

14. Get upset easily.

15. Have difficulty understanding abstract ideas.

16. Keep in the background.

17. Am not really interested in others.

18. Make a mess of things.

19. Seldom feel blue.

20. Do not have a good imagination.

(* Honesty-Humility of Brief HEXACO Inventory *: Modesty, sincerity, greed avoidance, fairness)

1. I consider myself an ordinary person and no better than others.

2. Even if I believe that flattering can lead to rewards, I would not do it.

3. For me, having a lot of money is not particularly important.

4. Even if something is of great value or a large amount, I would never accept a bribe.

5. I don’t want people to treat me as if I am better than them.

6. I would not pretend to like someone just to get them to do something for me.

7. I would like to live in a very expensive and upscale neighborhood.

8. If I knew I would never get caught, I would be tempted to steal one million yuan.

9. I believe I am more deserving of respect than the average person.

10. To get something from someone I don't like, I would pretend to be friendly to them.

11. I want others to see me driving a luxury car.

12. If I were short on money, I might not be able to resist the temptation to buy stolen goods.

13. I want others to know that I am an important person of high status.

14. If I want something from someone, I would laugh at their jokes even if they are not funny.

15. I would feel great joy if I had the opportunity to own expensive luxury items.

16. If I were sure I would not get caught, I would be tempted to use counterfeit money.

(* Psychological Entitlement Scale *: Entitlement)

1. I honestly feel I’m just more deserving than others.

2. Great things should come to me.

3. If I were on the Titanic, I would deserve to be on the first lifeboat!

4. I demand the best because I’m worth it.

5. **This is an attention check question. Please select the number three.**

6. I do not necessarily deserve special treatment.

7. I deserve more things in my life.

8. People like me deserve an extra break now and then.

9. Things should go my way.

10. I feel entitled to more of everything.

(* Interpersonal Reactivity Index-C *: Empathy)

1. I often feel tender-hearted and caring toward those who are less fortunate than I am.

2. Sometimes I am not very upset about other people’s misfortunes or problems.

3. I do get emotionally involved with the characters in novels.

4. I get worried and nervous and can’t calm down in emergencies.

5. When I watch plays or movies, I usually remain detached and don’t often get fully involved.

6. Before making decisions, I try to see where everyone is coming from in an argument.

7. When I see someone being taken advantage of, I feel an urge to protect them.

8. When I am in a highly emotional situation, I often feel helpless and at a loss.

9. Sometimes I try to imagine how things look from my friends’ point of view in order to understand them better.

10. It is rare for me to get fully involved in a good book or a good movie.

11. Other people’s misfortunes usually don’t upset me a great deal.

12. After seeing a play or a movie, I often feel as if I were one of the characters in it.

13. I get panicky and frightened when I am in a tense emotional situation.

14. When I see someone being treated unfairly, I sometimes don’t feel very sympathetic.

15. I believe that there are two sides to every question, so I often try to look at things from different points of view.

16. I consider myself a rather soft-hearted person.

17. When I see a good movie, I can easily identify with the feelings of a main character.

18. I get so nervous in emergencies that I can hardly control myself.

19. When I am angry with someone, I usually try to see things from his or her point of view.

20. When I read an interesting story or novel, I often imagine how I would feel if the events in it happened to me.

21. When I see someone in an accident who desperately needs help, I get so nervous that I almost fall apart.

22. Before criticizing someone, I try to imagine how I would feel if I were in his or her place.

Please respond to the following items using the number that best reflects your own beliefs. Please use the following 7-point scale:

1 = strong disagreement.

2 = moderate disagreement.

3 = slight disagreement.

4 = neither agreement nor disagreement.

5 = slight agreement.

6 = moderate agreement.

7 = strong agreement.

(* Power Scale *: Power)

1. I can get people to listen to what I say.

2. My ideas carry weight.

3. When I express my opinions, my views are influential.

4. I can get people to do things my way.

5. I feel that I have a great deal of power.

6. If I want to do something, I can usually manage to do it.

7. My views and ideas are often not ignored by others.

8. I can make decisions based on my own ideas.

(* Moral Grandstanding Motivation Scale *: Moral grandstanding)

1. I hope that my beliefs cause other people to want to share those beliefs.

2. I am particularly good at sharing my beliefs.

3. My beliefs should be inspiring to others.

4. I often share my beliefs in the hope of inspiring people to be more passionate about their beliefs.

5. When I share my beliefs, I do so to show people who disagree with me that I am better than them.

6. I share my beliefs to make people who disagree with me feel bad.

7. When I share my beliefs, I do so to shame people who do not share those beliefs.

8. When I share my beliefs, I do so in the hope that people different than me will feel ashamed of their beliefs.

9. I want to be on the right side of history about moral/political issues.

10. Even if expressing my views does not help anyone, it is important that I share them.

# Supplementary Material D.

# Instructions for Operation

**Instructions for the Typical Touch Force Test**

This test aims to collect the touch force of participants when typing on a keyboard and pressing an elevator button, using a film sensor system. After the test, the collected data will be combined with related questionnaire surveys to explore the potential connections between different touch forces.

The touch sensing system primarily operates using film sensors, an Arduino control board, and a signal conversion module. In this test, the device is used solely to collect touch force data and will not collect, detect, or record any other personal (private) information.

The results of the test will be used for research purposes only and will not be disclosed publicly. Thank you very much for your support and participation.

**Operation Instruction for the Touch Force Test**

Before the test, participants should ensure that their emotional and mental states are stable and normal, so that they can perform routine work tasks and study activities. If any unexpected situations occur, participants should promptly notify the researchers before the test begins. Additionally, prior to the formal start of the test, participants are required to complete the *Basic Information* under the guidance of the researchers to facilitate subsequent tests and surveys (in questionnaire form).

During the test, participants are only required to perform typing on the provided computer keyboard and pressing the elevator button in front of them. The total duration of the test is approximately 12 minutes (10 minutes for keyboard typing + 2 minutes for elevator button pressing).

Please pay attention to the following points:

1. Participants should type all the text displayed on the left side of the screen into the blank document on the right side using the keyboard. To ensure data accuracy, copying and pasting or any form of simplified input, including predictive text input, is strictly prohibited. Participants must use the default input method provided by the system to enter the characters.
2. Participants should press the elevator button a total of ten times, with a 5-second interval between each press.
3. Participants must ensure that the content they type matches the example text on the left side of the screen exactly.
4. During keyboard typing and elevator button pressing, participants should maintain a touch force similar to their daily habits. If any significant discomfort is felt, or if the film sensor affects their input behavior, participants should promptly communicate with the researchers and make appropriate adjustments.
5. There is no time limit or typing speed requirement for the test. Participants should complete the keyboard typing task based on their own capabilities. The purpose of the test is to evaluate the touch force distribution during typing and elevator button usage, not to pursue speed.

**Notes for Researchers**

Prior to the start of the test, researchers must first verify whether the test sensors are functioning properly to ensure the smooth progress of the test. This includes calibrating and testing the sensors to guarantee accurate data recording throughout the test.

During the test, researchers should closely monitor the participants’ input process to ensure that they are able to input text normally and accurately and complete the elevator button pressing operations as required.

Once the test is completed, researchers should promptly collect, save, and back up the test data. If the force data is lost or not properly recorded, they should communicate with the participant in a timely manner and reschedule the test. Proper management of the data is crucial for subsequent data analysis and the reliability of the research results.

**Sample contents of keyboard input (in Chinese Pinyin)**

Shenzhou shiba hao feixing chengzu 24 ri zai Jiuquan weixing fashe zhongxin shouci gongkai liangxiang, chengzu you hangtianyuan Ye Guangfu, Li Cong, Li Guangsu zucheng, Ye Guangfu danren zhiling zhang. Ye Guangfu shi Zhongguo di'er pi hangtianyuan, zhixing guo Shenzhou shisan hao zairen feixing renwu, Li Cong he Li Guangsu dou wei Zhongguo disan pi hangtianyuan, dou shi shouci zhixing feixing renwu, 3 ren dou wei "80 hou". Dangtian shi di jiu ge "Zhongguo hangtian ri", 54 nian qian de jintian, Zhongguo diyi ke renzao diqiu weixing "Dongfanghong yi hao" zai Jiuquan weixing fashe zhongxin fashe chenggong. Zai zhege teshu de rizi li, Shenzhou shiba hao 3 ming hangtianyuan zai zhe kuai Zhongguo hangtian shiye he "Liangdan yixing" jingshen de zhongyao faxiangdi yu zhongwai meiti jizhe jiti jianmian. Zhiling zhang Ye Guangfu zai jianmian hui shang xiang suoyou wei Zhongguo hangtian shiye wusi fengxian de fendouzhe, pan dengzhe zhijing.

Gen ju guanfang gongbu de jianli, Ye Guangfu yu 1980 nian 9 yue chusheng, jiguan Sichuan Chengdu, xian wei Zhongguo Renmin Jiefangjun hangtianyuan dadui yiji hangtianyuan, 2010 nian 5 yue ruxuan wei Zhongguo di'er pi hangtianyuan. 2021 nian 10 yue zhixing Shenzhou shisan hao zaizai feixing renwu, 2022 nian 6 yue bei shouyu "yingxiong hangtianyuan" rongyu chenghao, bing huo "san ji hangtian gongxun jiangzhang". Li Cong yu 1989 nian 10 yue chusheng, jiguan Hebei Handan, xian wei Zhongguo Renmin Jiefangjun hangtianyuan dadui si ji hangtianyuan, 2020 nian 9 yue ruxuan wei Zhongguo disan pi hangtianyuan. 2023 nian 12 yue, Hubei Xianning fasheng le yiqi huangjin qie'an, yi jia jin dian de gui tai bei qiao, lian baoxian gui ye bei zhuanye qiege gongju po huai, jin liu qian ke de huangjin shipin bei xijie yi kong.

2023 nian 12 yue 6 ri, shi weiyu Xianning Shi Tongchengxian de zhe jia jin dian kaizhang yingye de disan tian. Qingchen, qianlai shangban de dianyuan dakaimen, wu nei de changjing rang tamen da chi yi jing—wu nei yi pian langji, dianpu li daliang jinshi bei xijie yi kong.

# Supplementary Material E.

# Personality Traits

The Mini-International Personality Item Pool (Mini-IPIP) is a 20-item short form from the 50-item International Personality Item Pool—Five-Factor Model measure [3], which was initially developed by Donnellan et al. [4] and adapted to Chinese in Dong [5]. The Mini-IPIP consists of four items per Big Five trait (*extraversion*, *agreeableness*, *conscientiousness*, *neuroticism* and *openness*) and uses a 5-point scale (1 = “strongly disagree” to 5 = “strongly agree”) with consistent and acceptable internal consistencies.

We used the Honesty-Humility subscale from the 24-item Brief HEXACO Inventory (BHI) [6] on *modesty*, *sincerity*, *greed avoidance*, and *fairness* [7] and each contains 4 items with the scale from 1 (strongly disagree) to 5 (strongly agree).

To access *entitlement*, we implemented the Psychological Entitlement Scale (PES) originally developed in Campbell et al. [8] and adapted to Chinese [9]. The 9-item PES measures the extent of applicability regarding *entitlement* on a 5-point scale (1 = “very inapplicable” to 5 = “very applicable”). The scale is unidimensional and has been found to be reliable and valid [10,11] (α = 0.79).

*Empathy* was measured using a questionnaire Interpersonal Reactivity Index-C (IRI-C) [12,13], which measures empathy using 22 items. Participants were asked to rate the extent to which they (dis)agree with presented items about themselves on a 5-point Likert scale. The scale exhibited good reliability in all samples (α = 0.66).

To measure *power*, a modified and translated version of the classic Power Scale [14,15] was used. This scale is composed of 8 items, to which participants responded with their agreement on a 7-point scale.

We measured *moral grandstanding* using the modified 10-item Moral Grandstanding Motivation Scale [16], as it is a valid, adult-oriented and unidimensional measure. It uses a 7-point scale to investigate *moral grandstanding* motivations (α = 0.78).

All scales were administered in validated Chinese versions: Mini-IPIP, Brief HEXACO Inventory (BHI), Psychological Entitlement Scale, Interpersonal Reactivity Index-C (IRI-C), the classic Power Scale, and Moral Grandstanding Motivation Scale. Reverse-coded items were recoded before computing subscale scores. Furthermore, we conducted confirmatory factor analyses (CFA) for each multi-dimensional scale to verify their factor structures in our sample. The results, presented in Table S1, showed that all fit indices (CFI/TLI > 0.90; RMSEA/SRMR < 0.08) met acceptable psychometric standards [17], supporting the construct validity of these measures in our cultural context.

**Table S1**

Confirmatory factor analysis (CFA) fitting index of each scale.

| Scale name | χ² (df) | χ²/df | CFI | TLI | RMSEA [90% CI] | SRMR |
| --- | --- | --- | --- | --- | --- | --- |
| Mini-IPIP | 368.72 (160) | 2.30 | 0.92 | 0.90 | 0.07 [0.06, 0.08] | 0.06 |
| Brief HEXACO Inventory - Honesty-Humility | 85.10 (35) | 2.43 | 0.95 | 0.93 | 0.08 [0.06, 0.10] | 0.05 |
| Psychological Entitlement Scale | 45.25 (14) | 3.23 | 0.96 | 0.94 | 0.09 [0.06, 0.12] | 0.04 |
| Interpersonal Reactivity Index-C | 642.51 (344) | 1.87 | 0.93 | 0.92 | 0.06 [0.05, 0.07] | 0.07 |
| The classic Power Scale | 30.81 (9) | 3.42 | 0.97 | 0.95 | 0.09 [0.06, 0.13] | 0.03 |
| Moral Grandstanding Motivation Scale | 102.56 (54) | 1.90 | 0.95 | 0.94 | 0.06 [0.04, 0.08] | 0.05 |

# Supplementary Material F.

# Correlation Analysis and Reliability (α)

We focused on the correlation between all independent variables and the two dependent variables (keyboard typing force and elevator button touch force). The reliability coefficients (α) corresponding to each scale item are listed in Table S2. These coefficients are used to evaluate the internal consistency of the scales, with values closer to 1 indicating higher reliability. Generally, an α value above 0.6 is considered acceptable. In this study, the minimum α value (α_min_ = 0.62) exceeded 0.60, meeting the requirements of our discipline and indicating good internal consistency of the scales used.

Additionally, Fisher’s *r* to Z transformation is a method that converts Pearson’s correlation coefficient (*r*) into an approximately normally distributed value (z). This transformation is commonly used to compare differences between two correlation coefficients [18-20]. By employing this transformation, the differences in correlation coefficients between male and female samples can be calculated and represented using *p*-values (Table S2).

**Table S2**

Correlation for dependent variables and reliability

| Variable | α | Keyboard | | | |  | Elevator button | | | |
| --- | --- | --- | --- | --- | --- | --- | --- | --- | --- | --- |
|  |  | *r* | *r* (Male) | *r* (Female) | cor diff p |  | *r* | *r* (Male) | *r* (Female) | cor diff p |
| Force of touching keyboards | - | 1.00 | 1.00 | 1.00 | - |  | 0.07 | 0.06 | 0.07 | 0.98 |
| Force of touching elevator buttons | - | 0.07 | 0.06 | 0.07 | 0.98 |  | 1.00 | 1.00 | 1.00 | - |
| Age | - | 0.15 | 0.24 | 0.04 | 0.82 |  | 0.16 | 0.03 | 0.26 | 0.83 |
| BMI | - | 0.15 | 0.09 | 0.16 | 0.94 |  | **0.34**** | **0.45**** | 0.15 | 0.75 |
| Education level | - | 0.11 | 0.20 | 0.01 | 0.83 |  | 0.16 | 0.01 | 0.27 | 0.80 |
| Health status | - | 0.02 | 0.13 | -0.08 | 0.83 |  | -0.02 | 0.10 | -0.08 | 0.86 |
| Stress status | - | 0.14 | 0.12 | 0.20 | 0.93 |  | 0.14 | 0.23 | 0.08 | 0.89 |
| Sleep duration | - | 0.05 | 0.03 | 0.05 | 0.99 |  | -0.11 | -0.19 | -0.09 | 0.91 |
| Exercise frequency | - | 0.14 | 0.13 | 0.08 | 0.97 |  | **0.24**** | 0.20 | 0.22 | 1.00 |
| Monthly household income | - | 0.05 | 0.00 | 0.11 | 0.92 |  | -0.02 | 0.22 | -0.25 | 0.64 |
| Social stratification | - | -0.08 | 0.00 | -0.12 | 0.89 |  | 0.00 | 0.06 | -0.01 | 0.93 |
| *Extraversion* | 0.75 | -0.05 | -0.17 | 0.10 | 0.78 |  | **0.20*** | 0.25 | 0.20 | 0.97 |
| *Agreeableness* | 0.65 | -0.08 | **-0.29*** | 0.18 | 0.64 |  | -0.04 | -0.08 | 0.01 | 0.93 |
| *Conscientiousness* | 0.62 | -0.04 | -0.14 | 0.07 | 0.84 |  | 0.04 | -0.06 | 0.12 | 0.87 |
| *Neuroticism* | 0.70 | 0.18 | **0.28*** | 0.08 | 0.83 |  | 0.04 | 0.05 | 0.07 | 0.99 |
| *Openness* | 0.64 | -0.07 | -0.07 | -0.04 | 0.96 |  | -0.08 | -0.07 | -0.05 | 0.97 |
| *Modesty* | 0.63 | -0.02 | 0.19 | **-0.28*** | 0.63 |  | -0.01 | -0.19 | 0.11 | 0.78 |
| *Sincerity* | 0.74 | 0.03 | -0.02 | 0.06 | 0.94 |  | 0.07 | -0.10 | 0.18 | 0.76 |
| *Greed avoidance* | 0.75 | -0.02 | 0.05 | -0.14 | 0.86 |  | -0.13 | -0.20 | -0.05 | 0.90 |
| *Fairness* | 0.77 | -0.01 | **-0.26*** | **0.28*** | 0.59 |  | 0.08 | -0.08 | 0.23 | 0.75 |
| *Entitlement* | 0.79 | -0.10 | -0.16 | 0.03 | 0.85 |  | -0.16 | 0.04 | -0.27 | 0.76 |
| *Empathy* | 0.66 | 0.04 | 0.15 | -0.09 | 0.81 |  | -0.07 | -0.20 | 0.06 | 0.81 |
| *Power* | 0.84 | 0.06 | -0.17 | **0.31*** | 0.63 |  | 0.02 | 0.08 | -0.05 | 0.90 |
| *Moral grandstanding* | 0.78 | 0.10 | 0.07 | 0.11 | 0.97 |  | 0.00 | 0.19 | -0.24 | 0.67 |

Notes. α = internal consistency statistics (Cronbach’s alpha); cor diff *p* = correlation difference test (using Fischer’s r-to-z transformation) between sexes. Pearson correlation coefficients (r) are displayed between variables. **p* < 0.05, ***p* < 0.01, ****p* < 0.001.

**Table S3-1**

Exploratory sex-stratified Pearson correlations with keyboard touch force

| Variable | Males (N = 60) | | |  | | | Females (N = 55) | | | |  | |
| --- | --- | --- | --- | --- | --- | --- | --- | --- | --- | --- | --- | --- |
|  | *r* | *p* | *q* | |  | *r* | | *p* | *q* | *P* | |  |
| Age | 0.24 | 0.11 | 0.47 | |  | 0.04 | | 0.80 | 0.93 | 0.82 | |  |
| BMI | 0.09 | 0.55 | 0.78 | |  | 0.16 | | 0.28 | 0.63 | 0.94 | |  |
| Education level | 0.20 | 0.18 | 0.47 | |  | 0.01 | | 0.96 | 0.96 | 0.83 | |  |
| Health status | 0.13 | 0.39 | 0.66 | |  | -0.08 | | 0.60 | 0.83 | 0.83 | |  |
| Stress status | 0.12 | 0.43 | 0.66 | |  | 0.20 | | 0.18 | 0.63 | 0.93 | |  |
| Sleep duration | 0.03 | 0.84 | 0.88 | |  | 0.05 | | 0.76 | 0.93 | 0.99 | |  |
| Exercise frequency | 0.13 | 0.40 | 0.66 | |  | 0.08 | | 0.60 | 0.83 | 0.97 | |  |
| Monthly household income | 0.00 | 0.99 | 0.99 | |  | 0.11 | | 0.47 | 0.75 | 0.92 | |  |
| Social stratification | 0.00 | 0.99 | 0.99 | |  | -0.12 | | 0.43 | 0.75 | 0.89 | |  |
| *Extraversion* | -0.17 | 0.25 | 0.53 | |  | 0.10 | | 0.51 | 0.83 | 0.78 | |  |
| *Agreeableness* | -0.29 | 0.04 | **0.21** | |  | 0.18 | | 0.23 | 0.63 | 0.64 | |  |
| *Conscientiousness* | -0.14 | 0.34 | 0.62 | |  | 0.07 | | 0.64 | 0.83 | 0.84 | |  |
| *Neuroticism* | 0.28 | 0.04 | **0.21** | |  | 0.08 | | 0.60 | 0.83 | 0.83 | |  |
| *Openness* | -0.07 | 0.64 | 0.78 | |  | -0.04 | | 0.79 | 0.93 | 0.96 | |  |
| *Modesty* | 0.19 | 0.19 | 0.47 | |  | -0.28 | | 0.04 | **0.23** | 0.63 | |  |
| *Sincerity* | -0.02 | 0.92 | 0.92 | |  | 0.06 | | 0.70 | 0.83 | 0.94 | |  |
| *Greed avoidance* | 0.05 | 0.74 | 0.83 | |  | -0.14 | | 0.35 | 0.75 | 0.86 | |  |
| *Fairness* | -0.26 | 0.03 | **0.20** | |  | 0.28 | | 0.04 | **0.23** | 0.59 | |  |
| *Entitlement* | -0.16 | 0.28 | 0.53 | |  | 0.03 | | 0.84 | 0.93 | 0.85 | |  |
| *Empathy* | 0.15 | 0.32 | 0.62 | |  | -0.09 | | 0.55 | 0.83 | 0.81 | |  |
| *Power* | -0.17 | 0.25 | 0.53 | |  | 0.31 | | 0.03 | **0.21** | 0.63 | |  |
| *Moral grandstanding* | 0.07 | 0.63 | 0.78 | |  | 0.11 | | 0.50 | 0.75 | 0.97 | |  |

Notes. This table presents exploratory, hypothesis-generating analyses. Due to the high number of multiple comparisons, False Discovery Rate (FDR) correction (Benjamini-Hochberg procedure) was applied separately to the correlations within the male and female subgroups. The adjusted p-values (q-values) are the primary metric for interpretation. None of the correlations survived FDR correction at a threshold of q < 0.10. Bold q-values (q): These are the nominally significant (p < .05) correlations before correction. However, as the note above the table states, none are significant after FDR correction (all q > 0.10). Correlation Difference P-value (P): This p-value indicates whether the correlation coefficient (r) is significantly different between males and females. None of these are significant, meaning that while the strength of a correlation might look different between groups, these differences are likely due to chance.

**Table S3-2**

Exploratory sex-stratified Pearson correlations with elevator button touch force

| Variable | Males (N = 60) | | |  | | | Females (N = 55) | | | |  | |
| --- | --- | --- | --- | --- | --- | --- | --- | --- | --- | --- | --- | --- |
|  | *r* | *p* | *q* | |  | *r* | | *p* | *q* | *P* | |  |
| Age | 0.03 | 0.85 | 0.95 | |  | 0.26 | | 0.03 | 0.33 | 0.83 | |  |
| BMI | 0.45 | 0.01 | **0.14** | |  | 0.15 | | 0.45 | 0.60 | 0.75 | |  |
| Education level | 0.01 | 0.96 | 0.98 | |  | 0.27 | | 0.01 | 0.32 | 0.80 | |  |
| Health status | 0.10 | 0.51 | 0.72 | |  | -0.08 | | 0.10 | 0.78 | 0.86 | |  |
| Stress status | 0.23 | 0.12 | 0.33 | |  | 0.08 | | 0.23 | 0.78 | 0.89 | |  |
| Sleep duration | -0.19 | 0.19 | 0.42 | |  | -0.09 | | -0.19 | 0.76 | 0.91 | |  |
| Exercise frequency | 0.20 | 0.18 | 0.42 | |  | 0.22 | | 0.20 | 0.42 | 0.90 | |  |
| Monthly household income | 0.22 | 0.13 | 0.33 | |  | -0.25 | | 0.22 | 0.33 | 0.64 | |  |
| Social stratification | 0.06 | 0.69 | 0.81 | |  | -0.01 | | 0.06 | 0.97 | 0.93 | |  |
| *Extraversion* | 0.25 | 0.08 | 0.33 | |  | 0.20 | | 0.25 | 0.41 | 0.97 | |  |
| *Agreeableness* | -0.08 | 0.60 | 0.72 | |  | 0.01 | | -0.08 | 0.96 | 0.93 | |  |
| *Conscientiousness* | -0.06 | 0.70 | 0.81 | |  | 0.12 | | -0.06 | 0.71 | 0.87 | |  |
| *Neuroticism* | 0.05 | 0.74 | 0.81 | |  | 0.07 | | 0.05 | 0.78 | 0.99 | |  |
| *Openness* | -0.07 | 0.64 | 0.80 | |  | -0.05 | | -0.07 | 0.78 | 0.97 | |  |
| *Modesty* | -0.19 | 0.19 | 0.42 | |  | 0.11 | | -0.19 | 0.71 | 0.78 | |  |
| *Sincerity* | -0.10 | 0.51 | 0.72 | |  | 0.18 | | -0.10 | 0.42 | 0.76 | |  |
| *Greed avoidance* | -0.20 | 0.18 | 0.44 | |  | -0.05 | | -0.20 | 0.78 | 0.90 | |  |
| *Fairness* | -0.08 | 0.60 | 0.73 | |  | 0.23 | | -0.08 | 0.43 | 0.75 | |  |
| *Entitlement* | 0.04 | 0.80 | 0.87 | |  | -0.27 | | 0.04 | 0.33 | 0.76 | |  |
| *Empathy* | -0.20 | 0.18 | 0.43 | |  | 0.06 | | -0.20 | 0.76 | 0.81 | |  |
| *Power* | 0.08 | 0.60 | 0.72 | |  | -0.05 | | 0.08 | 0.78 | 0.90 | |  |
| *Moral grandstanding* | 0.19 | 0.19 | 0.43 | |  | -0.24 | | 0.19 | 0.36 | 0.67 | |  |

Notes. The same to Table S2-1.

# Supplementary Material G.

# VIF of Independent Variables

Taking the force of typing on the keyboard as examples, the VIF values for all the variables, as shown in Table S4, are below 5. This implies that the model does not exhibit any significant issue of multicollinearity. Consequently, the interpretation of the regression results related to participants’ touching force can be considered reliable and unaffected by multicollinearity concerns.

**Table S4**

Results of linear regression analysis.

| **Coefficient ^a^** | | | | | | | |
| --- | --- | --- | --- | --- | --- | --- | --- |
|  | Unstandardized coefficient | | Standardization coefficient |  |  | Covariance statistics | |
| Model | B | Standard error | Beta | *t* | Significance | Tolerance | VIF |
| (Constant) | 0.15 | 0.40 |  | 0.38 | 0.70 |  |  |
| *Extraversion* | -0.03 | 0.02 | -0.15 | -1.17 | 0.25 | 0.56 | 1.78 |
| *Agreeableness* | -0.01 | 0.03 | -0.05 | -0.44 | 0.66 | 0.65 | 1.54 |
| *Conscientiousness* | 0.00 | 0.02 | -0.02 | -0.20 | 0.84 | 0.74 | 1.35 |
| *Neuroticism* | 0.04 | 0.02 | 0.22 | 1.87 | 0.07 | 0.65 | 1.54 |
| *Openness* | -0.02 | 0.02 | -0.08 | -0.68 | 0.50 | 0.73 | 1.36 |
| *Modesty* | -0.03 | 0.03 | -0.13 | -0.82 | 0.42 | 0.36 | 2.80 |
| *Sincerity* | 0.01 | 0.02 | 0.04 | 0.33 | 0.74 | 0.52 | 1.91 |
| *Greed avoidance* | 0.00 | 0.02 | 0.01 | 0.09 | 0.93 | 0.71 | 1.40 |
| *Fairness* | -0.01 | 0.02 | -0.04 | -0.30 | 0.76 | 0.58 | 1.71 |
| *Entitlement* | -0.05 | 0.03 | -0.21 | -1.39 | 0.17 | 0.39 | 2.54 |
| *Empathy* | 0.00 | 0.04 | 0.01 | 0.09 | 0.93 | 0.75 | 1.34 |
| *Power* | 0.03 | 0.02 | 0.19 | 1.32 | 0.19 | 0.41 | 2.43 |
| *Moral grandstanding* | 0.02 | 0.02 | 0.13 | 0.95 | 0.34 | 0.46 | 2.16 |
| Sex | 0.02 | 0.04 | 0.09 | 0.61 | 0.54 | 0.46 | 2.18 |
| Age | 0.02 | 0.01 | 0.34 | 1.58 | 0.12 | 0.20 | 4.07 |
| BMI | 0.00 | 0.01 | 0.06 | 0.43 | 0.67 | 0.56 | 1.79 |
| Education level | -0.02 | 0.05 | -0.10 | -0.48 | 0.64 | 0.20 | 4.97 |
| Health status | 0.00 | 0.02 | 0.00 | 0.01 | 1.00 | 0.83 | 1.20 |
| Stress status | 0.01 | 0.02 | 0.08 | 0.65 | 0.52 | 0.59 | 1.68 |
| Sleep duration | 0.01 | 0.02 | 0.05 | 0.46 | 0.65 | 0.75 | 1.34 |
| Exercise frequency | 0.02 | 0.03 | 0.07 | 0.64 | 0.53 | 0.68 | 1.47 |
| Monthly household income | 0.01 | 0.02 | 0.09 | 0.75 | 0.46 | 0.61 | 1.65 |
| Social stratification | -0.02 | 0.01 | -0.16 | -1.28 | 0.20 | 0.56 | 1.77 |

1. Dependent variable: force of touching keyboards.

# Supplementary Material H.

# Participants’ Descriptive Statistics

**Table S5**

Descriptive statistics of the sample (N = 115).

| Variables | Mean (SD) or N (%) | Range |
| --- | --- | --- |
| **Demographic attributes** |  |  |
| Sex (Female) | 55 (47.83%) | / |
| Age | 23.11 (0.21) | 18 – 30 |
| BMI | 22.22 (0.29) | 15.63 – 32.32 |
| Education level (Undergraduate) | 32 (27.83%) | / |
| Health status (Fair) | 58 (50.43%) | / |
| Stress status (1-2 times a week) | 60 (52.17%) | / |
| Sleep duration (7-8 hours) | 54 (46.96%) | / |
| Exercise frequency (1-3 times per week) | 64 (55.65%) | / |
| Monthly household income (10,000 yuan to 14,999 yuan) | 34 (29.57) | / |
| Social stratification | 4.70 (0.12) | 2 – 8 |
| **Personality traits** |  |  |
| *Extraversion* | 2.93 (0.08) | 1.00 – 4.75 |
| *Agreeableness* | 3.65 (0.06) | 1.00 – 5.00 |
| *Conscientiousness* | 3.57 (0.07) | 1.75 – 5.00 |
| *Neuroticism* | 2.94 (0.07) | 1.00 – 4.75 |
| *Openness* | 3.52 (0.07) | 2.00 – 5.00 |
| *Modesty* | 3.26 (0.07) | 1.25 – 5.00 |
| *Sincerity* | 3.42 (0.08) | 1.25 – 5.00 |
| *Greed avoidance* | 2.72 (0.08) | 1.00 – 5.00 |
| *Fairness* | 3.95 (0.08) | 1.75 – 5.00 |
| *Entitlement* | 3.01 (0.06) | 1.44 – 4.56 |
| *Empathy* | 3.24 (0.03) | 2.45 – 4.45 |
| *Power* | 4.17 (0.09) | 2.00 – 6.63 |
| *Moral grandstanding* | 3.46 (0.08) | 1.40 – 5.20 |
| **Dependent variables (Criterion variables)** |  |  |
| Force of touching keyboards (N) | 0.68 (0.01) | 0.40 – 1.22 |
| Force of touching elevator buttons (N) | 1.54 (0.04) | 0.70 – 2.72 |

**Table S6**

Descriptive statistics for force outcomes (N).

| Variables | Mean (SD) | Median | IQR | Min | Max |
| --- | --- | --- | --- | --- | --- |
| Keyboard | 0.68 (0.04) | 0.66 | [0.65, 0.70] | 0.42 | 1.25 |
| Elevator button | 1.52 (0.20) | 1.49 | [1.38, 1.65] | 0.70 | 2.41 |

# References

1. Reinilä, E., Saajanaho, M., Fadjukoff, P., et al. The development of generativity in middle adulthood and the beginning of late adulthood: A longitudinal study from age 42 to 61. J. Adult. Dev. 2023; 30:291–304. https://doi.org/10.1007/s10804-022-09436-1
2. Linnemann, P., Wellmann, J., Berger, K., et al. Effects of age on trait resilience in a population-based cohort and two patient cohorts. J. Psychosom. Res. 2020; 136:110170. https://doi.org/10.1016/j.jpsychores.2020.110170
3. Goldberg, L.R. A broad-bandwidth, public-domain, personality inventory measuring the lower-level facets of several five-factor models. Pers. Psychol. Eur. 1999; 7:7–28.
4. Donnellan, M.B., Oswald, F.L., Baird, B.M., et al. The Mini-IPIP scales: Tiny-yet-effective measures of the Big Five factors of personality. Psychol. Assess. 2006; 18(2):192–203. https://doi.org/10.1037/1040-3590.18.2.192
5. Dong, Z. How to predict one’s personality trait by selfies on short video platforms [master’s thesis]. Wuhan: Wuhan Univ. 2020.
6. De, V.R.E. The 24-item Brief HEXACO Inventory (BHI). J. Res. Pers. 2013; 47(6):871–880. https://doi.org/10.1016/j.jrp.2013.09.003
7. Guo, Z., Li, W., Yang, Y., et al. Honesty-Humility and unethical behavior in adolescents: The mediating role of moral disengagement and the moderating role of system justification. J. Adolesc. 2021; 90:11–22. https://doi.org/10.1016/j.adolescence.2021.05.009
8. Campbell, W.K., Bonacci, A.M., Shelton, J., et al. Psychological entitlement: Interpersonal consequences and validation of a self-report measure. J. Pers. Assess. 2004; 83(1):29–45. https://doi.org/10.1207/s15327752jpa8301_04
9. Jiao, H. College students’ sense of power and prosocial behavior tendency: Mediation of psychological privilege and regulation of empathy [master’s thesis]. Shenyang: Shenyang Normal Univ. 2023.
10. Brunell, A.B. and Buelow, M.T. Homogenous scales of narcissism: Using the psychological entitlement scale, interpersonal exploitativeness scale, and narcissistic grandiosity scale to study narcissism. Pers. Individ. Dif. 2018; 123(1):182–190. https://doi.org/10.1016/j.paid.2017.11.025
11. Viskupič, F. and Wiltse, D.L. Psychological entitlement predicts support for mandatory COVID-19 vaccination. J. Med. Surg. Public. Health. 2024; 2:100043. https://doi.org/10.1016/j.glmedi.2023.100043
12. Zhan, Z. The relationship between grade, gender role, interpersonal orientation, and empathy. Institute of Education. National Taiwan Univ. 2020.
13. Zhang, F., Dong, Y., Wang, K. Reliability and validity of the Chinese version of the Interpersonal Reactivity Index-C. Chin J Clin Psychol. 2010; 18(2):155–157.
14. Anderson, C. and Galinsky, A.D. Power, optimism, and risk-taking. Eur. J. Soc. Psychol. 2006; 36(4):511–536. https://doi.org/10.1002/ejsp.324
15. Yao, Q., Wu, Z., Zhang, C., et al. Effect of power on conspicuous prosocial behavior. Acta. Psychol. Sin. 2020; 52(12):1421–1435. https://doi.org/10.3724/SP.J.1041.2020.01421
16. Grubbs, J.B., Warmke, B., Tosi, J., et al. Moral grandstanding in public discourse: Status-seeking motives as a potential explanatory mechanism in predicting conflict. PLoS One 14(10), 2019; e0223749. <https://doi.org/10.1371/journal.pone.0223749>
17. Hu, L.T. and Bentler, P.M. Cutoff criteria for fit indexes in covariance structure analysis: conventional criteria versus new alternatives. *Structural Equation Modeling: A Multidisciplinary Journal*. (1999) 6:1-55. <http://dx.doi.org/10.1080/10705519909540118>
18. Yu, T., Yu, G., Li, P.Y., et al. Citation impact prediction for scientific papers using stepwise regression analysis. Scientometrics 2014; 101:1233–1252. https://doi.org/10.1007/s11192-014-1279-6
19. Abdullah, A.S. Assessment of the risk factors of type II diabetes using ACO with self-regulative update function and decision trees by evaluation from Fisher’s Z-transformation. Med Biol Eng Comput. 2020; 60:1391–1415. https://doi.org/10.1007/s11517-022-02530-2
20. Jin, M., Ding, X., Han, H., et al. An improved method combining Fisher transformation and multiple endmember spectral mixture analysis for lunar mineral abundance quantification using spectral data. Icarus 2022; 380(1):115008. https://doi.org/10.1016/j.icarus.2022.115008
